# Integrative cross‐omics analysis identifies DNA methylation signatures associated with bilateral hippocampal volume, asymmetry and atrophy rate in the general population

**DOI:** 10.1002/alz70856_099862

**Published:** 2025-12-24

**Authors:** Dan Liu, Valentina Talevi, Juliana F. Tavares, Ruiqi Wang, Mohammed Aslam Imtiaz, Konstantinos Melas, Alexander Teumer, Wittfeld Katharina, Robert Francis Hillary, Dina Vojinović, Marian Beekman, Nicola Armstrong, Santiago Estrada, Henry Voelzke, Robin Bülow, Natalie Royle, Joanna M Wardlaw, Wei Wen, Perminder S. Sachdev, Karen A Mather, P. Eline Slagboom, Simon R Cox, Hans J. Grabe, Qiong Yang, N. Ahmad Aziz, Monique M.B. Breteler

**Affiliations:** ^1^ Population Health Sciences, German Center for Neurodegenerative Diseases (DZNE), Bonn, Germany; ^2^ German Center for Neurodegenerative Diseases (DZNE), Bonn, North Rhine‐Westphalia, Germany; ^3^ German Center for Neurodegenerative Diseases (DZNE), Bonn, Germany; ^4^ Boston University, Boston, MA, USA; ^5^ DZHK (German Center for Cardiovascular Research), Greifswald, Germany; ^6^ Institute for Community Medicine, University Medicine Greifswald, Greifswald, Germany; ^7^ University Medicine Greifswald, Greifswald, Germany; ^8^ German Center for Neurodegenerative Disease (DZNE) Rostock/ Greifswald, Greifswald, Germany; ^9^ University of Edinburgh, Edinburgh, United Kingdom; ^10^ Department of Epidemiology, Erasmus University Medical Center, Rotterdam, Netherlands; ^11^ Molecular Epidemiology, Department of Biomedical Data Sciences, Leiden University Medical Center, Leiden, Netherlands; ^12^ Curtin University, Perth, Western Australia, Australia; ^13^ AI in Medical Imaging, German Center for Neurodegenerative Diseases (DZNE), Bonn, Germany; ^14^ German Centre for Cardiovascular Research (DZHK), Greifswald, Germany; ^15^ University Medicine Greifswald, Greifswald, Mecklenburg‐Voor‐Pommeren, Germany; ^16^ IXICO, London, United Kingdom; ^17^ University of Edinburgh and UK DRI, Edinburgh, United Kingdom; ^18^ Centre for Healthy Brain Ageing, University of New South Wales, Sydney, NSW, Australia; ^19^ Centre for Healthy Brain Ageing (CHeBA), UNSW Sydney, Sydney, NSW, Australia; ^20^ Centre for Healthy Brain Ageing (CHeBA), University of New South Wales, UNSW Sydney, NSW, Australia; ^21^ Leiden University Medical Center, Leiden, Netherlands; ^22^ Lothian Birth Cohort Studies, Edinburgh, Scotland, United Kingdom; ^23^ University of Edinburgh, Edinburgh, Scotland, United Kingdom; ^24^ German Centre for Neurodegenerative Diseases (DZNE), Greifswald, Germany; ^25^ Boston University School of Public Health, Boston, MA, USA; ^26^ The Framingham Heart Study, Framingham, MA, USA; ^27^ Faculty of Medicine, University of Bonn, Bonn, North Rhine‐Westphalia, Germany; ^28^ Institute for Medical Biometry, Informatics and Epidemiology (IMBIE), Faculty of Medicine, University of Bonn, Bonn, Germany

## Abstract

**Background:**

The hippocampus, a critical brain structure for learning and memory, exhibits left‐right volumetric differences (LHCV and RHCV) and asymmetry. However, the molecular underpinnings of these features remain largely unknown in the general population.

**Method:**

We performed a meta‐analysis of epigenome‐wide association studies (EWAS) across six population‐based cohorts (8,156 individuals, 53% women, mean age: 60.7 years, age range: 30 to 92 years) to identify blood‐based methylation signatures associated with LHCV, RHCV, and hippocampal asymmetry. Integrative cross‐omics analyses using individual‐level genetic, methylation, and gene expression data from Rhineland Study participants (*n* > 2,624) was employed to elucidate the underlying molecular mechanisms. We further evaluated the relationships between ten dietary patterns, baseline methylation signatures, and longitudinal changes in hippocampal volumes and asymmetry.

**Result:**

Distinct CpG sites and differentially methylated regions (DMRs) for LHCV, RHCV, and hippocampal asymmetry converged on pathways related to neuronal differentiation and development. Integrative analyses identified 15 CpG/DMR‐gene expression pairs associated with LHCV and 18 pairs with RHCV, implicating immnue‐related (i.e. *LGALS3BP and TREM5)* and synaptic function genes *(i.e. MIR181A1HG and CORO1B*). Baseline methylation at these loci was significantly associated with rates of bilateral hippocampal loss over time, explaning over 10% of the variation in hippocampal atrophy rates. Four CpGs were consistently associated with hippocampal asymmetry cross‐sectionally and longitudinally, with sex‐specific differences. Moreover, higher adherence to healthy dietary patterns was associated with these methylation signatures.

**Conclusion:**

Our findings reveal methylation signatures as potential biomarkers or therapeutic targets for age‐ or neurodegeneration‐related hippocampal atrophy. They further underscore the influence of modifiable dietary patterns on methylation and hippocampal health.

